# Short-term oral rapamycin prevents age-related learning impairment in mice

**DOI:** 10.31491/apt.2020.09.033

**Published:** 2020-09-29

**Authors:** Haoyi Lei, Juan Wang, Warren Ladiges, Zhou Jiang

**Affiliations:** a Department of Comparative Medicine, School of Medicine, University of Washington, Seattle, WA, USA.

**Keywords:** Age-related learning impairment, rapamycin, aging mice

## Abstract

Effective treatments to prevent or delay age-related learning impairment are not generally available. In a preliminary preclinical study, mice 20 months of age were fed a diet containing 14 ppm rapamycin, an inhibitor of mTOR, for three months and then tested in a spatial navigation task. Mice fed the nonmedicated control diet showed learning impairment while mice fed the rapamycin diet were not learning impaired. This observation provides support for additional preclinical studies and suggests that short-term rapamycin treatment could be a possible strategy for preventing or delaying age-related cognitive impairment in people.

Learning impairment can develop with increasing age due to changes in brain structure and function and the development of age-related diseases [1]. Effective treatments to prevent or delay age-related learning impairment are not generally available, but recent work with rapamycin, an allosteric inhibitor of mTOR, showed the ability to prevent sleep deprived learning impairment when given parenterally to aging mice [2].

In a follow up study as reported here, rapamycin (Southwest Research Institute) was given orally at a dose of 14 ppm in the chow (prepared by TestDiet, Inc) to 20-month old female C57BL/6 mice for three months. Mice were then tested using the Box maze, a spatial navigation task that assesses learning ability [3]. The study was approved by the University of Washington IACUC.

The data in (Figure 1) show that over the course of three months, female mice from 20 months of age to 23 months of age fed a nonmedicated control chow diet showed learning impairments as they aged. In contrast, mice fed the rapamycin diet over the same time period were not learning impaired and had a learning ability similar to younger mice. There was no difference in food consumption between the two groups.

This observation suggests that further studies are warranted to investigate the effectiveness and mechanism of short-term rapamycin treatment as a possible strategy for preventing or delaying age-related cognitive impairment.

## Figures and Tables

**Figure 1.**
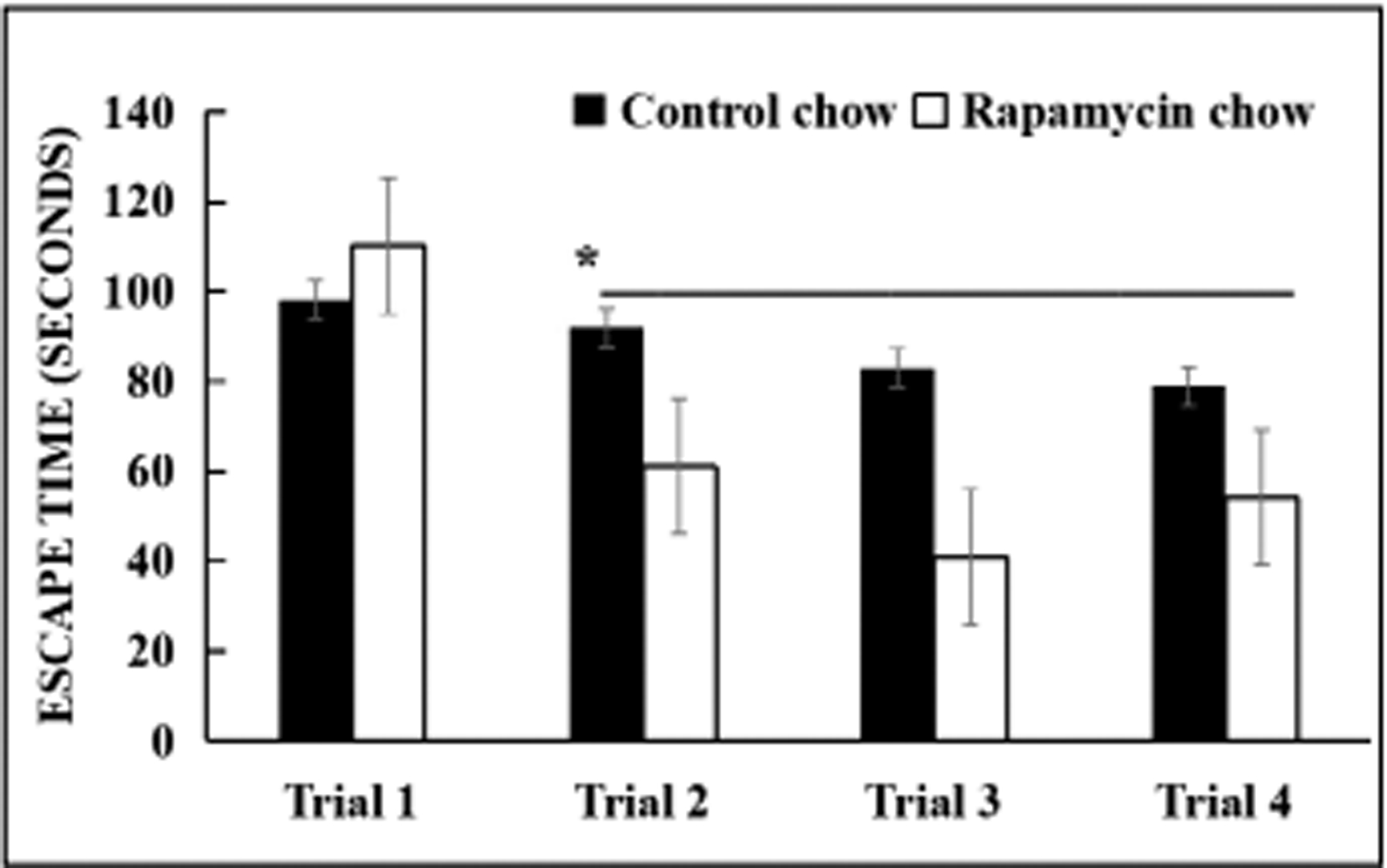
Female C57BL/6 mice, 20 months of age, were fed a rodent chow diet containing rapamycin (14 ppm) or a control diet (nonmedicated rodent chow) for three months and then tested in a spatial navigation task. Learning ability was measured by how quickly each mouse could find an escape hole over four trials. Mice fed the nonmedicated diet were learning impaired as shown by the inability to learn where the escape hole was. Mice fed the rapamycin diet quickly learned where the escape hole was by the second trial, and this ability was maintained for trials 3 and 4. *N* = 20 mice per cohort. * Significance was at *P* < 0.05, using student’s t test and SEM to compare the two groups for each trial.

## References

[1] Daniel L The Impact of age on cognition. Pubmed Central Journal, 2015, 36(3): 111–121. 10.1055/s-0035-1555115 PMC4906299 27516712

[2] Mukherjee K, Lee A, Zhu L, Sleep-deprived cognitive impairment in aging mice is alleviated by rapamycin. Aging Pathobiology and Therapeutics, 2019, 1(1): 05–09. 10.31491/apt.2019.12.002 PMC8789090 35083443

[3] Darvas M, Mukherjee K, Lee A, A Novel One-Day Learning Procedure for Mice. Current Protocols in Mouse Biology, 2020, 10(1): e68. 32096920 10.1002/cpmo.68

